# Design, Development, and Validation of a Chatbot to Support Health Care Professionals Experiencing Workplace Aggression: Protocol for a Mixed Methods Study

**DOI:** 10.2196/92511

**Published:** 2026-06-18

**Authors:** Alba García-Viola, Verónica V Márquez-Hernández, José Miguel Garrido-Molina, Alfredo Alcayde-García, Mª Carmen Rodríguez-García

**Affiliations:** 1 Andalusian Health Service Almería, Andalusia Spain; 2 University of Almería Almería, Andalusia Spain; 3 Centro de Emergencias Sanitarias 061 Almería, Andalusia Spain

**Keywords:** chatbot, digital health intervention, health care professionals, violence, workplace aggression

## Abstract

**Background:**

Workplace violence against health care professionals has increased worldwide, leading to negative psychological, professional, and organizational outcomes. Despite existing prevention and reporting programs, underreporting and lack of accessible, confidential support persist. Digital health tools, including chatbots, may offer scalable support, guidance, and follow-up for affected professionals.

**Objective:**

This study aims to design, develop, and validate a chatbot (*Sanidad Segura*) to assist health care professionals who experience workplace aggression and evaluate its usability, readability, and exploratory indicators of perceived usefulness and support in a pilot study.

**Methods:**

This study will follow a mixed methods design conducted in two main phases: (1) design, development, and content validation of the chatbot based on literature review, institutional protocols, and expert consensus; and (2) pilot-testing, including usability and readability assessment using standardized instruments, as well as feasibility and acceptability evaluation among health care professionals working in emergency and critical care settings in Almería (Spain). The study is aligned with the Medical Research Council framework for complex interventions, incorporating development and feasibility stages. Quantitative data will be collected using the System Usability Scale and Inflesz readability scale. Qualitative data will be collected through semistructured interviews and analyzed using thematic analysis to explore user experience and identify barriers to and facilitators of use.

**Results:**

The study has been funded for a 2-year period starting on December 18, 2024. Quantitative outcomes will include usability scores (System Usability Scale), readability scores (Inflesz), and participants’ sociodemographic characteristics. Qualitative findings will identify themes related to usability, user experience, and suggestions for improvement. Integration of quantitative and qualitative findings will be conducted through triangulation to provide a comprehensive understanding of the usability, acceptability, and readability of the chatbot.

**Conclusions:**

This study addresses the increasing incidence of workplace violence against health care professionals through the development of a new chatbot (*Sanidad Segura*). This intervention seeks to facilitate the identification, support, and follow-up of affected individuals while minimizing the adverse effects of such events on their physical and psychological well-being, social interaction, and professional performance. *Sanidad Segura* will enable confidential case reporting and provide access to tailored medical, psychological, and legal resources, as well as information about institutional support services. This project represents a crucial step toward implementing an integrated digital framework for the detection, management, and prevention of workplace violence in health care settings.

**International Registered Report Identifier (IRRID):**

PRR1-10.2196/92511

## Introduction

Workplace violence constitutes a global public health problem particularly prevalent in settings characterized by high levels of interpersonal interaction, with the health care sector being the most affected [1,2]. In this context, the situation is especially concerning due to the sustained increase in violent incidents over the past decade, with an even more pronounced rise during the recent COVID-19 pandemic [3,4].

The consequences of workplace violence extend beyond the individual, affecting health care workers’ well-being, patient safety, and team dynamics, ultimately reducing the quality of services provided by health care systems [5,6]. Among health care professionals, exposure to workplace violence has been associated with higher levels of burnout, stress, and psychological trauma, as well as decreased job satisfaction [7-9], which, in turn, translates into a greater intention to leave the job [9]. Moreover, workplace violence generates significant costs for health care systems by increasing absenteeism, staff turnover, and the need for replacement personnel, thereby negatively impacting productivity and the economic resources of health care institutions [10,11].

Violence in health care settings may be physical, verbal, or psychological, with the latter being the most frequent and least visible forms [12,13]. Despite their high prevalence, a large number of workplace violence incidents are not formally reported or recorded in official reporting systems, limiting accurate knowledge of their true magnitude and hindering the implementation of preventive and supportive measures [6,14,15].

The problem is particularly acute in emergency departments, which are consistently identified as high-risk settings for workplace violence. Previous studies have reported high rates of verbal and physical aggression among emergency health care professionals, with verbal aggression being the most prevalent form and frequently underreported [16,17]. Global evidence also indicates a higher prevalence of workplace violence in emergency and psychiatric settings than in other hospital services [13,18,19].

In Spain, workplace violence against health care professionals represents a well-documented and growing public health concern. Studies conducted in hospital settings report a high prevalence of aggression, particularly affecting nursing staff and frontline professionals, with nonphysical forms such as verbal and psychological abuse being the most frequent [20,21]. This problem is especially evident in high-demand areas such as emergency departments, where exposure to aggression is common, often involves patients and relatives, and remains substantially underreported [9,13,20].

Within the Andalusian Health Service (*Servicio Andaluz de Salud*), specific protocols for the prevention of and response to aggression against health care professionals have been developed [22]. These institutional measures are currently implemented in the study setting, namely, the Almería Health District, where health care professionals working in emergency and critical care units have access to formal reporting systems, legal support, and preventive protocols. However, aggressive incidents continue to occur, and their psychological impact is not always addressed in an early and systematic manner. This situation highlights the need for complementary tools that facilitate immediate and confidential access to guidance, psychological support, and self-care strategies, contributing to a comprehensive approach to workplace violence in health care settings and reinforcing the effectiveness of existing protocols [13,23].

In this context, the development of innovative digital solutions aimed at supporting health care professionals who have experienced workplace aggression emerges as a promising strategy. Conversational agents based on structured and rule-based architectures have demonstrated utility in health care by providing information, guidance, and emotional support in an accessible and continuous manner [24-26]. There is recent evidence suggesting that this type of technological assistance can be effectively integrated into workplace violence prevention programs, enhancing their reach and effectiveness [26].

However, currently available tools are not usually specifically designed to address workplace violence in health care settings from a comprehensive, professional-centered perspective. Within this framework, *Sanidad Segura* has been developed as a pioneering technological tool aimed at bridging this gap by integrating assistance, emotional support, and referral to legal and psychological resources, offering accessible, safe, and complementary support to existing institutional measures against violence toward health care professionals.

The development of *Sanidad Segura* is guided by the updated Medical Research Council framework for complex interventions. A preliminary program theory underpins the intervention, whereby chatbot components (emotional support, legal guidance, incident reporting, and psychoeducational content) are expected to improve perceived support, reduce barriers to reporting, and enhance help-seeking behaviors. *Sanidad Segura* is designed as a postincident support tool intended to be used after exposure to workplace aggression rather than during acute violent situations. The chatbot provides structured guidance on emotional coping strategies, incident documentation, legal rights, and access to institutional and psychological support services.

The platform operates through a structured conversational workflow based on predefined modules. After accessing the chatbot, users are guided through a sequence of options tailored to their needs following a workplace aggression incident. These modules include (1) emotional support and validation, (2) guidance on how to document and report the incident, (3) information on legal rights and institutional procedures, and (4) access to psychological and occupational support resources. Users interact with the chatbot by selecting options or entering short text inputs, which are categorized using predefined intents and routed through decision tree pathways. The chatbot does not generate free-text responses autonomously; instead, it delivers standardized, expert-validated content adapted to user input.

This study aims to design, develop, and validate this chatbot (*Sanidad Segura*) to assist health care professionals who experience workplace aggression and evaluate its usability, readability, and exploratory indicators of perceived usefulness and support in a pilot study.

## Methods

### Study Design

This study follows a two-phase mixed methods design: (1) design, development, and content validation; and (2) pilot feasibility, usability, and acceptability study. These phases correspond to the development and feasibility stages described in the Medical Research Council framework for complex interventions.

### Study Setting and Participants

Participants will be recruited from health care professionals currently active in emergency and critical care units in the Almería Health District (Spain). Eligibility will be restricted to individuals engaged in active clinical practice within the designated units. Professionals currently enrolled in any formal structured intervention targeting workplace aggression—whether psychological, legal, or organizational in nature—will be excluded. This criterion is intended to prevent potential confounding between ongoing therapeutic or remedial processes and the experimental chatbot exposure, thereby preserving the internal validity of the usability and acceptability assessments.

Sample size estimation for this pilot study was primarily guided by the requirements of the planned summative usability evaluation rather than by hypothesis testing for clinical efficacy outcomes—an approach consistent with current methodological guidance for pilot and feasibility studies of digital health technologies [27,28]. For summative usability assessments using standardized instruments such as the System Usability Scale (SUS), Sauro and Lewis [29] recommend samples of between 40 and 100 participants to achieve margin-of-error estimates of –5 to 10 points to +5 to 10 points at the 95% confidence level, thereby enabling clinically interpretable and psychometrically reliable scoring. Sample sizes within this range also provide sufficient statistical power to detect moderate between-group differences in SUS scores (ie, ≥5 points assuming an SD of approximately 15) and to generate stable estimates of acceptability and perceived usefulness across professional subgroups [29,30]. For pilot feasibility studies specifically targeting digital health interventions, precedent in the published literature supports the use of samples of 40 to 70 participants to ensure adequate precision in the estimation of key design parameters for future confirmatory trials [27,31,32].

Applying these methodological frameworks to the specific population under study and using a finite population correction formula with a 95% confidence level and –5% to +5% precision margin, a minimum of 178 participants is required from an eligible population of 330 professionals. This calculation ensures both representative coverage of the target population and sufficient variability in usability and acceptability responses across clinical roles (physicians, registered nurses, and nursing assistants). Accounting for an anticipated 20% attrition rate, 223 participants will be invited to take part. This sample size substantially exceeds the minimum thresholds recommended for summative usability evaluation and pilot feasibility studies [27,29,31] and, therefore, is considered appropriate to achieve the dual objectives of this study: generating robust usability and acceptability data and informing the design of a subsequent full-scale evaluation.

### Procedure

#### Phase 1: Chatbot Design, Development, and Content Validation

The design of the chatbot will be guided by a review of the literature, protocols, and guidelines on the prevention and management of aggression. In addition, a multidisciplinary expert panel will be established comprising health care professionals, lawyers, national police officers and civil guards, psychologists, and social workers with experience in aggression cases. The content validity index proposed by Lawshe [33] will be used to evaluate the relevance, comprehensibility, and clarity of each item as assessed by the expert panel. Content validity will include both item-level and scale-level indexes, with an expert panel of 12 members. An average content validity index of 0.8 or higher will be considered indicative of good content validity. Additionally, the modified κ coefficient will be calculated to determine the proportion of agreement regarding item relevance, with values of κ of 0.60 or higher considered acceptable.

During chatbot development, both software engineers and health care professionals will collaborate to ensure that the tool meets users’ needs. The chatbot will be accessible online, maintain confidentiality, and be designed for intuitive use. The chatbot will follow a hybrid rule-based conversational architecture based on predefined decision trees informed by institutional protocols and expert consensus.

Natural language processing techniques will be used to categorize user inputs into predefined intent categories, allowing the chatbot to guide users through structured conversational pathways based on decision tree logic. The chatbot will guide the user through a structured pathway including emotional validation, assessment of immediate needs, provision of coping strategies, and step-by-step instructions for incident reporting. Depending on user input, the system adapts responses within predefined decision trees to ensure consistency and safety.

The development will incorporate an approach centered on empathy and emotional understanding, providing supportive, validating, and nonjudgmental responses directed toward health care professionals. The chatbot will deliver practical information and resources to assist users in managing aggressive situations effectively.

The empathetic approach of the chatbot is operationalized through predefined supportive language patterns, including validation of emotional experiences, normalization of reactions to workplace aggression, and encouragement to seek appropriate support. This empathy is directed exclusively toward the health care professional and does not involve adaptive emotional inference beyond predefined categories.

During this phase, the focus will be on chatbot development and content validation. Technical performance aspects such as system functionality, response consistency, and alignment with institutional protocols will be internally tested by the research team and expert panel to ensure readiness for pilot evaluation. Crisis escalation pathways will be implemented. In cases of high-risk content, the chatbot will provide emergency contact information and recommend immediate human support.

#### Phase 2: Pilot-Testing

A pilot study will be conducted using a convenience sample of health care professionals described in the Study Setting and Participants section to evaluate the usability and feasibility of the chatbot. Following approval from the ethics committee, eligible participants will receive an email invitation detailing the study objectives, procedures, and ethical considerations. Participants who provide informed consent will be granted access to the chatbot.

This phase aims to assess usability, readability, and overall user experience through a mixed methods approach combining quantitative and qualitative data collection. This study follows the SPIRIT (Standard Protocol Items: Recommendations for Interventional Trials) and CONSORT-EHEALTH (Consolidated Standards of Reporting Trials of Electronic and Mobile Health Applications and Online Telehealth) guidelines.

### Quantitative Measures

#### Sociodemographic Variables

Data will be collected on sex, age, workplace setting, professional category, years of professional experience, length of time in the current unit, and prior experience of workplace aggression (current or past violence survivor).

#### Readability Assessment

Chatbot responses will be evaluated using the Inflesz scale [26], a validated instrument for assessing readability in Spanish texts. Scores range from 0 to 100 and are categorized as follows: very difficult (<40), somewhat difficult (40-55), standard (55-65), fairly easy (65-80), and very easy (>80).

#### Usability Assessment

Usability will be measured using the SUS [34,35], a validated 10-item questionnaire assessing perceived usability across dimensions such as learnability, efficiency, and satisfaction. Each item is rated on a 5-point Likert scale. Final scores range from 0 to 100, with higher scores indicating better usability. Scores will be interpreted as follows: poor (<60), marginal (60-69), acceptable (70-79), good (80-89), and excellent (≥90).

### Qualitative Data Collection

Qualitative data will be collected through semistructured interviews conducted after chatbot use focusing on user experience, perceived usefulness, emotional impact, and suggestions for improvement, as well as through open-ended survey responses embedded within the usability questionnaire. To better understand participants’ experiences with the chatbot, the interviews will include open and conversational questions such as “Can you tell me how you felt while using the chatbot?” “Was there any part that you found especially helpful or unhelpful?” “Did the information and responses seem clear to you?” “Did you encounter any difficulties while interacting with the chatbot?” and “What would you change or improve if you could?” An interview guide will be developed to ensure consistency across participants while still allowing for flexibility to explore emerging topics and deepen relevant aspects of the experience. These questions are intended to encourage participants to speak freely about their perceptions of usability and the usefulness of the intervention.

### Qualitative Analysis

A reflexive thematic analysis will be conducted following the methodology of Braun and Clarke [36]. The analysis will proceed through the following phases: (1) data familiarization, (2) initial code generation, (3) theme development, (4) theme review, (5) theme definition and naming, and (6) reporting.

Two independent researchers will code the data inductively. A coding framework will be iteratively developed and refined through regular meetings. Discrepancies will be resolved through discussion and consensus and, if necessary, consultation with a third researcher. Data management and coding will be supported by qualitative analysis software. To enhance rigor, strategies such as investigator triangulation, audit trails, and reflexivity will be used.

### Mixed Methods Integration

**Figure 1:**
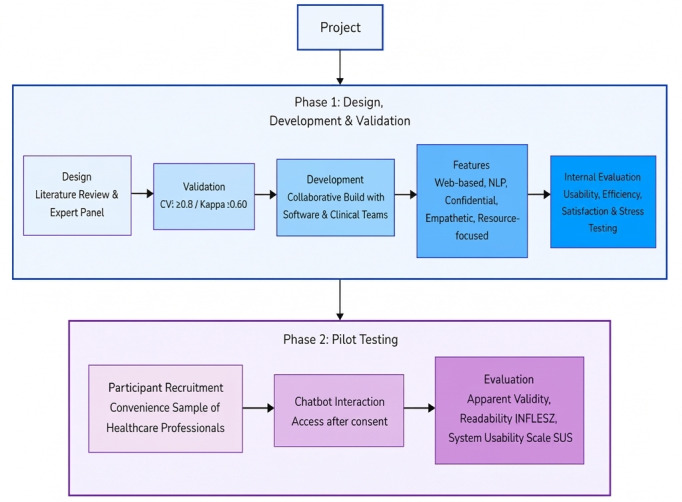
Mixed methods study design for the development and pilot validation of the Sanidad Segura chatbot for health care professionals exposed to workplace aggression in Almería, Spain. CVI: content validity index; NLP: natural language processing; SUS: System Usability Scale.

A convergent mixed methods design will be used (Figure 1). Quantitative and qualitative data will be collected concurrently, analyzed separately, and then integrated during interpretation.

Integration will be conducted through triangulation to compare findings across data sources; joint displays to visually integrate quantitative and qualitative results; and examination of convergence, complementarity, and discrepancies.

This approach will provide a comprehensive understanding of chatbot usability, acceptability, and readability, ensuring that quantitative findings are contextualized and enriched by qualitative insights.

### Ethical Considerations

The study will be performed in accordance with good clinical practice and the Declaration of Helsinki. This study received approval from the Provincial Ethics Committee of Almería (code: 132/2024). Health care professionals will participate in the study on a voluntary basis and may withdraw at any time without providing justification and without any impact on their legal rights. All participants will receive clear and detailed information regarding the study’s objectives, procedures, and potential implications and must provide informed consent prior to inclusion.

The *Sanidad Segura* chatbot will request explicit consent before collecting, processing, or transmitting any personal data. The information collected will be used exclusively for the purposes described in the study protocol and for the management of aggression in accordance with established institutional procedures.

Participant confidentiality and anonymity will be maintained at all times. Personal data will be processed in compliance with Regulation (EU) 2016/679 of the European Parliament and the Organic Law 3/2018 on the Protection of Personal Data and Guarantee of Digital Rights. For analysis purposes, data will be handled in an anonymized manner so that no participant can be identified.

Safety considerations include escalation pathways to human services in cases of high-risk disclosures.

### Statistical Analysis

Quantitative data will be analyzed using SPSS (version 30; IBM Corp). A descriptive analysis will be performed for all variables. Continuous variables will be summarized using means and SDs (or medians and IQRs where appropriate), whereas categorical variables will be presented as frequencies and percentages. Normality will be assessed using the Kolmogorov-Smirnov test. Depending on data distribution, parametric tests (Student *t* test and one-way ANOVA) or nonparametric equivalents (Mann-Whitney *U* test and Kruskal-Wallis test) will be applied to compare groups. Associations between categorical variables will be examined using the chi-square test. A significance level of a *P* value of less than .05 will be adopted.

### Data Management

Chatbot interaction data will be stored in secure encrypted servers compliant with the General Data Protection Regulation. Data will be anonymized and structured into a database including interaction logs, categorized queries, and use patterns. Access will be restricted to the research team.

### Protocol Registration

This study protocol has been registered at ClinicalTrials.gov (identifier: NCT07473531). The registered protocol includes detailed information on study design, methodology, and planned analyses, ensuring transparency and reproducibility.

## Results

The study has been funded for a 2-year period starting on December 18, 2024. This study will generate both quantitative and qualitative outcomes to evaluate the chatbot. Quantitative outcomes will include usability scores measured using the SUS and readability scores assessed using the Inflesz scale, along with participants’ sociodemographic characteristics.

Data collection was conducted between February and May 2026. A final convenience sample of 105 participants was obtained. At the time of manuscript submission, data analysis had been completed using SPSS Statistics (version 30.0; IBM Corp). The analyses included descriptive statistics, group comparisons, and correlation analyses as part of the pilot study. The results are expected to be published in an open-access journal in September 2026, with a potential second publication derived from the qualitative analysis anticipated in 2027. Qualitative findings will be derived from semistructured interviews, allowing for an in-depth exploration of user experience. Thematic analysis will be used to identify key themes related to usability, user experience, and suggestions for improvement.

Finally, quantitative and qualitative findings will be integrated through a convergent mixed methods approach using triangulation techniques to provide a comprehensive understanding of the usability, acceptability, and readability of the chatbot.

## Discussion

Workplace violence against health care professionals remains a critical and escalating global challenge. Recent evidence indicates that exposure to verbal threats, physical assaults, and psychological aggression continues to rise, with substantial negative consequences for health care workers’ mental health, job satisfaction, professional retention, and quality of patient care [25]. Studies conducted over the last years consistently report associations between workplace violence and increased levels of anxiety, depression, burnout, and reduced organizational commitment [10].

The growing awareness of workplace violence as a critical threat for health care professionals underscores the need for innovative, accessible, and evidence-based strategies to support those affected [2]. Despite the implementation of institutional prevention programs and legal frameworks, underreporting persists, largely due to fear of retaliation; normalization of violence; and lack of accessible, confidential reporting mechanisms. This study will focus on the design, development, and validation of *Sanidad Segura*, a chatbot developed to assist health care professionals who experience workplace aggression, and evaluate its usability, readability, and exploratory indicators related to perceived usefulness and support in a pilot study. Recent studies suggest that conversational agents can provide timely guidance, psychoeducational support, and facilitate help-seeking behaviors, particularly in contexts where time constraints and stigma limit access to conventional support pathways [25,37].

*Sanidad Segura* is designed to function as both an individual support tool and an organizational intelligence resource. At the individual level, the chatbot will enable confidential identification and reporting of aggression-related incidents while providing tailored medical, psychological, and legal guidance. Early access to structured support following exposure to violence has been shown to mitigate long-term psychological distress and reduce the likelihood of chronic mental health issues [11]. The interactive and anonymous nature of chatbot-based interventions may lower barriers to disclosure, particularly among professionals who would otherwise refrain from formal reporting. From an organizational perspective, integrating *Sanidad Segura* into existing occupational health frameworks may strengthen the culture of safety and reporting. There is evidence from recent health care management studies suggesting that institutions with proactive reporting systems and transparent response mechanisms demonstrate improved staff trust, reduced turnover intention, and enhanced patient safety outcomes [10]. In addition, *Sanidad Segura* is expected to provide valuable information on workplace violence in health care, thereby supporting organizational learning and prevention strategies. On the basis of notification records (reported events), *Sanidad Segura* can generate aggregated insights into the incidence and prevalence of workplace aggression. These data enable the identification of vulnerable professional groups and high-risk clinical settings, thereby informing targeted prevention strategies, training initiatives, and more efficient resource allocation. This evidence will address the lack of careful planning, resource management, and ongoing evaluations to ensure effective implementation and sustain impact in reducing workplace violence across health care settings [38], and it will provide empirical support for institutional and legislative decision-making related to workplace violence prevention.

The methodological design of this study emphasizes usability, readability, and acceptability as critical prerequisites for effective digital health implementation. Previous studies evaluating health care chatbots indicate that high usability scores are strongly linked to sustained engagement and perceived usefulness, underscoring the relevance of usability for the successful adoption of digital health interventions [25]. In fact, the use of validated instruments such as the SUS is consistent with current best practices in digital intervention development and aligns with the tools used in other comparable studies to assess usability [39,40].

This is the first study in the country aiming to design, develop, and validate a chatbot supporting health care professionals facing workplace aggression. This protocol represents a significant step toward the development of an integrated digital framework for the detection, management, and prevention of workplace violence in health care settings. By combining individual support with organizational data, *Sanidad Segura* has the potential to reduce the burden of workplace aggression, improve professional well-being, and inform evidence-based prevention strategies. If effective, *Sanidad Segura* could be integrated into the health care system and scaled to other regions nationwide. Future studies will be required to evaluate effectiveness, long-term impact, and scalability across diverse health care contexts.

This study is positioned as a feasibility and usability study rather than a full effectiveness evaluation. Qualitative findings complement the limitations of traditional usability instruments such as the SUS. Safety considerations are addressed through controlled design and escalation pathways.

It is important to note that *Sanidad Segura* is not based on generative artificial intelligence models but rather on a controlled rule-based system, which enhances safety, predictability, and alignment with validated institutional protocols.

## Abbreviations

CONSORT-EHEALTH: Consolidated Standards of Reporting Trials of Electronic and Mobile Health Applications and Online Telehealth
SPIRIT: Standard Protocol Items: Recommendations for Interventional Trials
SUS: System Usability Scale

## Data Availability

The datasets generated or analyzed during this study are available from the corresponding author on reasonable request.
